# Comparative safety profile of tyrosine kinase inhibitors in NSCLC: a network meta-analysis of hypertension and thrombotic risks

**DOI:** 10.3389/fphar.2025.1491990

**Published:** 2025-01-29

**Authors:** Mingming Tan, Chenwei Pu, Zhenzhen Wang, Chengwei Jin

**Affiliations:** ^1^ Department of Respiratory and Critical Care Medicine, Zibo Central Hospital, Zibo, China; ^2^ Department of Infectious Diseases, Zibo Central Hospital, Zibo, China; ^3^ Department of Cardiology, Zibo Central Hospital, Zibo, China

**Keywords:** non-small cell lung cancer, tyrosine kinase inhibitors, hypertension, thrombotic events, network meta-analysis, NSCLC

## Abstract

**Background:**

This study examines the risks of hypertension and thrombotic events in NSCLC patients treated with Tyrosine Kinase Inhibitors (TKIs).

**Objective:**

To compare the safety profiles of TKIs used in NSCLC treatment, focusing on hypertension and thrombotic risks.

**Methods:**

A comprehensive search identified randomized controlled trials evaluating the effects of TKIs in NSCLC patients. Bayesian network meta-analysis was employed to construct a comparative network of treatments.

**Results:**

Thirty studies involving 11,375 patients were included. Erlotinib had the lowest incidence of hypertension (SUCRA: 91.1%), followed by chemotherapy (88.8%). For thrombotic events, Erlotinib had the lowest risk (SUCRA: 66.1%), while Anlotinib and Cabozantinib had the highest thrombotic risks (SUCRA: 26.9%).

**Conclusion:**

Erlotinib presents the lowest risk for hypertension and thrombotic events, making it a preferred choice for NSCLC patients with cardiovascular concerns.

**Systematic review registration:**

https://www.crd.york.ac.uk/prospero, identifier CRD42024530770.

## Introduction

Lung cancer remains the leading cause of cancer-related mortality worldwide, with non-small cell lung cancer (NSCLC) comprising 80%–90% of primary lung malignancies. For patients with stage IV NSCLC, the standard treatment typically involves chemotherapy and palliative radiation therapy. Despite advancements in treatment options, including molecular targeted therapies and immunotherapy, the overall 5-year survival rate for stage IV NSCLC remains dismally low at 4%–6% (David et al., 2017).

Research has underscored the critical role of vascular endothelial growth factor (VEGF) in tumor growth, progression, and metastasis, primarily by promoting angiogenesis (Apte et al., 2019). Targeting the VEGF signaling pathway has become a cornerstone in the development of anticancer therapies. Bevacizumab, a VEGF receptor tyrosine kinase inhibitor (VEGFR-TKI), effectively neutralizes VEGF, inhibiting the tumor’s blood supply and thereby showing significant clinical efficacy across various cancers, including breast cancer, colorectal cancer, and NSCLC (Al Kawas et al., 2022; Ahluwalia et al., 2014; Cardones and Banez, 2006). Similarly, epidermal growth factor receptor (EGFR)-targeted therapies, such as cetuximab, have improved the prognosis for lung cancer patients (Le et al., 2021).

Despite the therapeutic benefits of antiangiogenic agents, these drugs are associated with increased risks of arterial thrombotic events and hemorrhagic complications. While hypertension represents another frequent adverse event, it can typically be managed with conventional antihypertensive medications (Krupitskaya and Wakelee, 2009). However, the precise magnitude of cardiovascular risks, particularly hypertension and thrombotic events, associated with antiangiogenic targeted therapies in NSCLC remains inadequately characterized (Castel et al., 2011).

Therefore, a comprehensive meta-analysis of contemporary randomized controlled trials could provide more robust evidence regarding the cardiovascular safety profile of antiangiogenic therapies in NSCLC, with particular emphasis on hypertensive and thrombotic complications.

## Methods

### Literature search

A comprehensive search was conducted using the following terms: (“EGFR-TKI” OR “VEGF-TKI” OR “Gefitinib” OR “Erlotinib” OR “Icotinib” OR “Afatinib” OR “Dacomitinib” OR “Osimertinib” OR “ALK inhibitors” OR “Brigatinib” OR “Lorlatinib” OR “Alectinib”) AND (“NSCLC” OR “non-small-cell lung carcinoma” OR “non-small cell lung cancer”). Our search covered published articles from electronic databases, including PubMed, Embase, and the Cochrane Library, up to 1 June 2024. Additionally, we manually searched abstracts from the American Society of Clinical Oncology and the World Congress on Lung Cancer to identify unpublished studies and ongoing clinical trials. Only studies published in English were included, and we also hand-searched the references of the included studies.

### Inclusion criteria

Studies were eligible if they compared tyrosine kinase inhibitors (TKIs) combined with chemotherapy or other treatments versus TKIs alone. The criteria for inclusion were (David et al., 2017): prospective randomized controlled trials (RCTs) comparing TKIs alone or in combination with chemotherapy in NSCLC patients (Apte et al., 2019); reported data on the number of patients with hypertension or thrombotic adverse reactions, as well as the total number of patients with adverse events; and (Al Kawas et al., 2022) original articles published in English. Exclusion criteria included (David et al., 2017): single-arm clinical trials (Apte et al., 2019); case reports or review articles; and (Al Kawas et al., 2022) clinical trials with fewer than 10 participants.

### Data extraction

Data extracted from each study included the year of publication, first author, trial name, patient demographics (age, sex), ECOG score, disease status, smoking history, type of TKIs used, incidence of hypertension and thrombotic events, total number of subjects, and follow-up duration. Data extraction, study design, and results were reviewed by two independent reviewers. Disagreements were resolved through discussion, and if consensus was not reached, a third independent reviewer was consulted. Data were standardized according to pre-specified criteria to ensure consistency across studies. Data extraction was performed independently by two reviewers. In cases of discrepancies between reviewers, a third reviewer was consulted, and a consensus was reached through discussion. When necessary, we contacted the original authors for clarification or additional data. This process ensured the accuracy and completeness of the extracted data.

### Risk of bias assessment

Two researchers independently assessed the risk of bias using the Cochrane Handbook tool, evaluating the following domains: (David et al., 2017): random sequence generation, (Apte et al., 2019), allocation concealment, (Al Kawas et al., 2022), blinding of participants and personnel, (Ahluwalia et al., 2014), completeness of outcome data (Cardones and Banez, 2006), selective reporting, and (Le et al., 2021) other potential sources of bias. Trials were categorized into three levels: high risk, low risk, and unclear risk (Higgins et al., 2011).

### Data analysis

Randomized controlled trials (RCTs) conducted across various institutions frequently yield heterogeneous efficacy outcomes, challenging the establishment of definitive therapeutic hierarchies. Network meta-analysis emerges as a valuable methodological approach to facilitate comprehensive comparisons among diverse therapeutic agents evaluated in different RCTs. In this systematic review and network meta-analysis, we sought to evaluate and compare the cardiovascular safety profiles of various treatment strategies, specifically focusing on hypertensive and thrombotic risks in patients with non-small cell lung carcinoma. The surface under the cumulative ranking curve (SUCRA) probability was employed to establish a hierarchical ranking of therapeutic strategies based on their cardiovascular safety profiles (Sonbol et al., 2020). Statistical analysis was performed using R (version 4.2.1) with the gemtc and rjags packages. We used odds ratios (OR) with 95% confidence intervals (CI) for dichotomous adverse reaction data. Network meta-analysis (NMA) and Bayesian aggregation were conducted using Markov Chain Monte Carlo (MCMC) simulations (Moher et al., 2015). Funnel plots, generated with Stata (version 15.0), assessed potential bias in network comparisons (Salanti et al., 2011). Stata also produced network diagrams depicting hypertension occurrences as an adverse event. These diagrams visually represent evidence, with nodes indicating different interventions and connecting lines showing direct comparisons. The size of each node and line width are proportional to the number of cases (Chaimani et al., 2013). The treatment effect was summarized using the surface under the cumulative ranking curve (SUCRA), where a higher SUCRA value indicates a better treatment effect (Daly et al., 2019). To assess the robustness of our findings, we conducted sensitivity analyses by excluding studies with high risk of bias. Additionally, we performed subgroup analyses based on patient characteristics and treatment duration to explore potential sources of heterogeneity. These analyses helped to evaluate the consistency of our results across different study conditions and patient populations.

## Results

### Study selection

**FIGURE 1:**
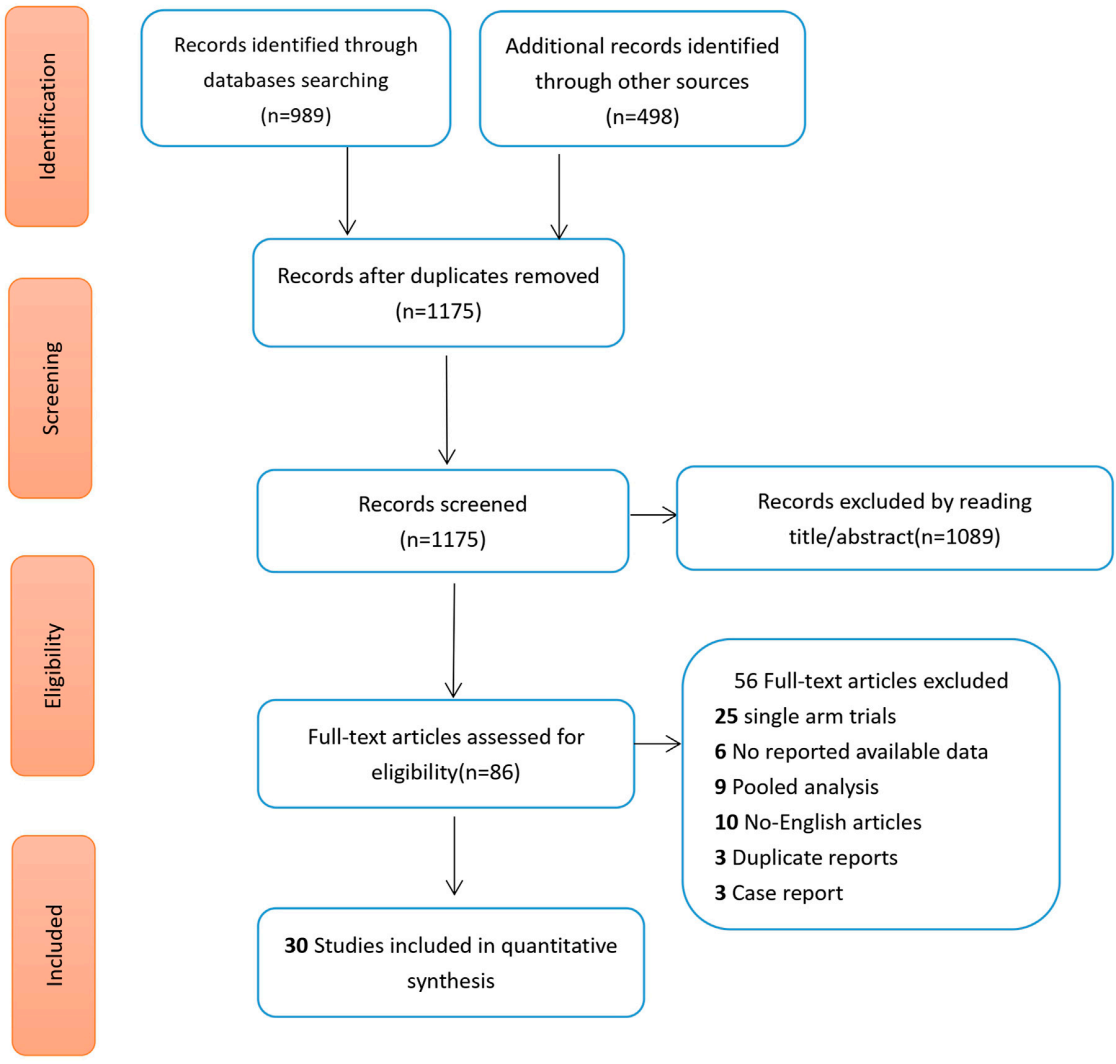
Literature screening flow chart.

Following an extensive search, a total of 30 randomized controlled trials (RCTs) were included, involving 11,375 non-small cell lung cancer (NSCLC) patients treated with tyrosine kinase inhibitors (TKIs). Eleven vascular-targeted drugs were compared, focusing primarily on adverse events such as hypertension and thrombotic events (venous and arterial thrombosis). Figure 1 illustrates the search process: initially, 1,487 articles containing the search terms were identified. After removing duplicates, 86 articles were selected for full-text review based on their titles and abstracts. Ultimately, 30 RCTs were chosen based on their randomization methodology and the relevance of their outcome measures (Table 1) (Nakagawa et al., 2019; Han et al., 2018; Garon et al., 2014; Akamatsu et al., 2021; Ramlau et al., 2012; Ninomiya et al., 2023; Piccirillo et al., 2022; Liu et al., 2021; Kato et al., 2018; Besse et al., 2017; Zhao et al., 2021; Sun et al., 2018; Spigel et al., 2018; Cortot et al., 2020; Wakelee et al., 2017; Tiseo et al., 2017; Hanna et al., 2016; Karayama et al., 2016; Neal et al., 2016; Baggstrom et al., 2017; O'Brien et al., 2015; Pujol et al., 2015; Doebele et al., 2015; Twelves et al., 2014; Natale et al., 2011; Paz-Ares et al., 2012; Johnson et al., 2013; Herbst et al., 2011; Spigel et al., 2011; Heymach et al., 2008).

**TABLE 1:** Baseline characteristics of included studies.

First author	Year	Registration number	Control arm treatment	Patients in control arm (n)	Age	Male (%)	Disease stage	ECOG
Nakagawa et al. (2019)	2019	RELAY	Erlotinib	225	64 (56–70)	83 (37%)	Stage IV 189 (84%) Other 36 (16%)	= 0 119 (53%) = 1 106 (47%)
Han et al. (2018)	2018	ALTER 0303	Placebo	143	≤60 (62.9%) 61–69 (28.7%) ≥70 (8.4%)	97 (67.8%)	IIIB 7 (4.9%) IV 136 (95.1%)	= 0 22 (15.4%) = 1 120 (83.9%) = 2 1 (0.7%)
Garon et al. (2014)	2014	REVEL	Placebo plus docetaxel	625	61 (25–86)	415 (66%)	NA	= 0 199 (32%) = 1 425 (68%)
Akamatsu et al. (2021)	2021	UMIN000023761	Osimertinib	41	68 (43–82)	17 (41)	IIIB 2 (5) IV26 (63) Recurrence13 (32)	= 0 17 (42) = 1 24 (58)
Ramlau et al. (2012)	2012	NCT00532155	Placebo + Docetaxel	457	59.6 (27–80)	300 (65.6)	I II 43 (9.4) III 135 (29.6) IV 265 (58.0)	= 0 151 (33.0) = 1 283 (61.9) = 2 23 (5.0)
Ninomiya et al. (2023)	2023	jRCTs061180006	afatinib	50	71.0 (32–84)	22 (44.0)	III B 1 (2.0) IV 38 (76.0)	= 0 28 (56.0) = 1 22 (44.0)
Piccirillo et al. (2022)	2022	BEVERLY	Erlotinib	80	67.7 (60.7–73.6)	30 (37.5)	IIIB 5 (6.3) IV 75 (93.8)	= 0 47 (58.8) = 1 29 (36.3) = 2 4 (5.0)
Liu et al. (2021)	2021	NA/ALTER 1202?	Placebo	15	59 (43–75)	11 (73.3)	NA	= 1 13 (86.7) = 2 2 (13.3)
Kato et al. (2018)	2018	JO25567	erlotinib	77	67.0 (60–73)	26 (34%)	IV 62 (81%) Postoperative recurrence 15 (19%)	= 0 41 (53%) = 1 36 (47%)
Besse et al. (2017)	2017	IFCT-0703	Placebo	71	61 (44–71)	45 (63%)	IA 59 (83) IB 12 (17)	= 0 58 (82) = 1 13 (18)
Zhao et al. (2021)	2021	ACTIVE	Placebo Plus Gefitinib	156	60 (51–65)	62 (39.7)	IIIB 8 (5.1) IV 148 (94.9)	= 0 50 (32.1) = 1 105 (67.3)
Sun et al. (2018)	2018	KCSG-LU12-07	Placebo	47	67 (50–83)	43 (91.5%)	NA	= 0 3 (6.4%) = 1 44 (93.6%)
Spigel et al. (2018)	2018	NCT00892710	Pemetrexed	48	72 (51–84)	30 (63)	IIIB 5 (10) IV 43 (90)	NA
Cortot et al. (2020)	2020	IFCT-1103	Docetaxel	55	59.7 (35.8; 78.9)	42 (76.4%)	NA	= 0–1 51 (92.8%)
Wakelee et al. (2017)	2017	E1505	chemotherapy	749	61 (IQR 55,67)	375 (50%)	I (27) II (42) III (31)	NA
Tiseo et al. (2017)	2017	FARM6PMFJM	cisplatin and etoposide chemotherapy regimen	103	63 (41–81)	70 (68)	NA	= 0 57 (55.3) = 1 35 (34) = 2 11 (10.7)
Hanna et al. (2016)	2016	LUME-Lung 2	Placebo + pemetrexed	360	59 (26–86)	208 (57.8)	Stage < IIIB 69 (19.2) Stage IIIB 52 (14.4) Stage IV 239 (66.4)	= 0 139 (38.6) = 1 221 (61.4)
Karayama et al. (2016)	2016	NA	Pemetrexed maintenance	55	66 (50–75)	39 (70.9)	IIIB 7 (12.7) IV 48 (87.3)	= 0 48 (87.3) = 1 7 (12.7)
Neal et al. (2016)	2016	ECOG-ACRIN 1512	Erlotinib/Cabozantinib	38/38	66.3 ± 9.8/65.9 ± 10.1	18 (47)/14 (37)	IV M1a 8 (21)/6 (16) IV M1b 21(55)/18 (47) Recurrent 9 (24)/14 (37)	= 0 9 (24)/9 (24) = 1 24 (63)/25 (66) = 2 5 (13)/4 (11)
Baggstrom et al. (2017)	2017	CALGB 30607	Placebo	104	66.3 ± 9.3	60 (57.7%)	IIIB 12 (11.5%) IV 92 (88.5%)	= 0 42 (40.4%) = 1 62 (59.6%)
O'Brien et al. (2015)	2015	EORTC 08092	Placebo	52	64.6 (25.9–80.7)	25 (48.1)	NA	= 0 11 (21.2) = 1 39 (75.0) = 2 2 (3.8)
Pujol et al. (2015)	2015	IFCT-0802	chemotherapy	37	60.1 (46–72)	26 (70.3%)	NA	= 0–1 35 (94.6%) = 2 2 (5.4%)
Doebele et al. (2015)	2015	NCT01160744	pemetrexed and carboplatin	71	18 to <65 years 37 (52.1) ≥65 years 34 (47.9)	45 (63.4)	NA	= 0–1 65 (91.5) = 2 4 (5.6)
Twelves et al. (2014)	2014	NCT00600821	Axitinib + paclitaxel/carboplatin	58	61.7	36 (62.1)	IIIB 6 (10.3) IV 52 (89.7)	= 0 16 (27.6) = 1 42 (72.4)
Natale et al. (2011)	2011	NCT00364351	vandetanib	623	61 (26–92)	381 (61)	IIIb 106 (17) IV 517 (83)	= 0 194 (31) = 1 363 (58) = 2 65 (10)
Paz-Ares et al. (2012)	2012	NA	placebo + gemcitabine + cisplatin	387	58 (22–77)	245 (63.3)	IIIB 47 (12.1) IV 340 (87.9)	= 0 143 (37.0) = 1 244 (63.0)
Johnson et al. (2013)	2013	ATLAS	Bevacizumab	373	64 (23–83)	196 (53)	IIIb 37 (10) IV 310 (83) Recurrent 25 (7)	= 0 173 (47) = 1 198 (53) = 2 1 (0.3)
Herbst et al. (2011)	2011	NCT00130728/BeTa	erlotinib	317	65	170 (54%)	NA	= 0 121 (38%) = 1 176 (56%) = 2 20 (6%)
Spigel et al. (2011)	2011	SALUTE	Placebo	50	64 (47–82)	30 (60%)	NA	= 0 23 (46) = 1 21 (42) = 2 6 (12)
Heymach et al. (2008)	2008	NA	paclitaxel and carboplatin	52	59 (42–83)	37 (71%)	IIIB 5 (10) IV 47 (90)	= 0 16 (31) = 1 36 (69)

Smoking status	Experimental arm treatment	Patients in experimental arm (n)	Age	Male (%)	Disease stage	ECOG	Smoking status	Median follow-up (month)
Ever 73 (32%) Never 139 (62%) Unknown 13 (6%)	Ramucirumab +erlotinib	224	65 (57–71)	83 (37%)	Stage IV 195 (87%) Other 29 (13%)	= 0 116 (52%) = 1 108 (48%)	Ever 64 (29%) Never 134 (60%) Unknown 26 (12%)	20·7 months (IQR 15·8–27·2)
Once or now smoking 77 (53.8%) Non-smoker 66 (46.2%)	Anlotinib	294	≤60 (52.0%) 61–69 (42.5%) ≥70 (5.4%)	188 (64.0%)	IIIB 15 (5.1%) IV 277 (94.2%) Other 2 (0.7%)	= 0 59 (20.1%) = 1 233 (79.3%) = 2 2 (0.7%)	Once or now smoking 143 (48.6%) Non-smoker 151 (51.4%)	NA
Ever 483 (77%) Never 141 (23%) Unknown 1 (<1%)	Ramucirumab plus docetaxel	628	62 (21–85)	419 (67%)	NA	= 0 207 (33%) = 1 420 (67%)	Ever 518 (82%) Never 109 (17%) Unknown 1 (<1%)	9.5 months [IQR 4·4–14·9]
Never 20 (49) Smoker or former smoker 21 (51)	Osimertinib + bevacizumab	40	70 (41–82)	16 (40)	IIIB 2 (5) IV33(83) Recurrence 5 (12)	= 0 20 (50) = 1 20 (50)	Never 21 (53) Smoker or former smoker 19 (48)	16.0 (2.4–22.6)
NA	Aflibercept + Docetaxel	456	59.6 (27–84)	305 (66.9)	I-II 36 (7.9) III 125 (27.4) IV 284 (62.3)	= 0 149 (32.7) = 1 286 (62.7) = 2 21 (4.6)	NA	23.0 months
NA	afatinib plus bevacizumab	49	69.0 (48–83)	22 (44.9)	III B 2 (4.1) IV 37 (75.5)	= 0 32 (65.3) = 1 17 (34.7)	NA	24 months
Never 37 (46.3) Former/current 34 (42.5)	Erlotinib + bevacizumab	80	65.9 (57.9–71.8)	28 (35.0)	IIIB 3 (3.8) IV 77 (96.3)	= 0 52 (65.0) = 1 26 (32.5) = 2 2 (2.5)	Never 46 (57.5) Former/current 34 (42.5)	36.3 months (95% CI: 30.7–40.9)
Never 4 (26.7) Former 11 (73.3)	Anlotinib	27	60 (31–70)	19 (70.4)	NA	= 0 1 (3.7) = 1 24 (88.9) = 2 2 (7.4)	Never 11 (40.7) Former 15 (55.6) Current 1 (3.7)	11 months
Never smoker 45 (58%) Former light smoker 6 (8%) Other 26 (34%)	erlotinib plus bevacizumab	75	67.0 (59–73)	30 (40%)	IIIB 1 (1%) IV 60 (80%) Postoperative recurrence 14 (19%)	= 0 43 (57%) = 1 32 (43%)	Never smoker 42 (56%) Former light smoker 9 (12%) Other 24 (32%)	20.4 months (IQR 17.4–24.1)
Never 6 (8) Current/former 64 (92)	Pazopanib	71	57 (33–70)	41 (58)	IA 54 (76) IB 16 (24)	= 0 47 (66) = 1 24 (34)	Never 6 (8) Current/former 65 (92)	47 months (range 0.3–66 months)
Nonsmoker 121 (77.6) Smoker 35 (22.4)	Apatinib Plus Gefitinib	157	57 (51–65)	66 (42.0)	IIIB 5 (3.2) IV 152 (96.8)	= 0 48 (30.6) = 1 107 (68.2)	Nonsmoker 115 (73.2) Smoker 42 (26.8)	15.8 months (interquartile range 12.6–20.4 months)
Current or ex-smoker 41 (87.2%) Never smoker 6 (12.8%)	Pazopanib	48	66.5 (57–79)	40 (83.3%)	NA	= 0 1 (2.1%) = 1 47 (97.9%)	Current or ex-smoker 43 (89.6%) Never smoker 5 (10.4%)	30.1 months
Former smoker 26 (54) Current smoker 20 (42) Lifetime nonsmoker 2 (4)	Pemetrexed and Bevacizumab/Pemetrexed, Bevacizumab, and Carboplatin	63/61	72 (50–90)/73 (48–90)	36 (57%)/34 (56%)	IIIB 4 (6)/2 (3) IV 58 (92)/59 (97)	NA	Former smoker 44 (70)/42 (70) Current smoker 16 (25)/13 (21) Lifetime nonsmoker 3 (5)/6 (10)	NA
Never smokers 9 (16.4%)	Paclitaxel plus bevacizumab	111	59.6 (18.6; 81.8)	78 (70.3%)	NA	= 0–1 103 (92.8%)	Never smokers9 (8.1%)	36.2 months (range: 28.6; 43.0),
NA	chemotherapy plus bevacizumab	752	61(IQR 54,67)	371 (49%)	I (25) II (45) III (29)	NA	NA	50·3 months (IQR 32.9–68.0)
NA	cisplatin + etoposide + bevacizumab	101	64 (45–79)	69 (68.3)	NA	= 0 53 (52.5) = 1 42 (41.6) = 2 6 (5.9)	NA	34.9 months (interquartile range, 22.5–41.5 months)
Current smoker 44 (12.2) Ex-smoker 194 (53.9) Never smoker 122 (33.9)	Nintedanib + pemetrexed	353	60 (21–84)	195 (55.2)	Stage < IIIB 57 (16.1) Stage IIIB 77 (21.8) Stage IV 219 (62.0)	= 0 135 (38.2) = 1 218 (61.8)	Current smoker 51 (14.4) Ex-smoker 193 (54.7) Never smoker 109 (30.9)	19.4 months (interquartile range [IQR] = 13.6–26.9)
Never smoker 13 (23.6) Former smoker 27 (49.1) Current smoker 15 (27.3)	Pemetrexed and bevacizumab maintenance	55	65 (39–75)	35 (63.6)	IIIB 6 (10.9) IV 47 (85.5)	= 0 50 (90.9) = 1 5 (9.1)	Never smoker 19 (34.5) Former smoker 20 (36.4) Current smoker 16 (29.1)	24.1 months (range; 12.7–47.1)
Current 8 (21)/9 (24) Former 25 (66)/23 (61) Never 5 (13)/6 (16)	Erlotinib + Cabozantinib	35	63.5 ± 9.0	18 (51)	IV M1a 5 (14) IV M1b 20 (57) Recurrent 10 (29)	= 0 8 (23) = 1 23 (66) = 2 4 (11)	Current 8 (23) Former 21 (60) Never 6 (17)	17.0 months
Nonsmoker 10 (9.6%) Past smoker 67 (64.4%) Current smoker 27 (26.0%)	Sunitinib	106	63.6 ± 10.0	57 (53.8%)	IIIB 14 (13.2%) IV 92 (86.8%)	= 0 40 (37.7%) = 1 66 (62.3%)	Nonsmoker 5 (4.7%) Past smoker 76 (71.7%) Current smoker 25 (23.6%)	20.6 months, with a range of 6.3–60.9 months
Never 10 (19.2) Past 35 (67.3) Current 4 (7.7)	Pazopanib	50	64.2 (28.4–81.1)	21 (42.0)	NA	= 0 18 (36.0) = 1 32 (64.0)	Never 11 (22.0) Past 26 (52.0) Current 11 (22.0)	13.4 months
NA	Chemotherapy + bevacizumab	37	61.2 (43–75)	25 (67.6%)	NA	= 0–1 33 (89.2%) = 2 3 (8.1%)	NA	37.7 months (25–50 months)
Never smoked or smoked <100 cigarettes16 (22.5)	pemetrexed and carboplatin + ramucirumab	69	18 to <65 years 37 (53.6) ≥65 years 32 (46.4)	36 (52.2)	NA	= 0–1 64 (92.8) = 2 3 (4.3)	Never smoked or smoked <100 cigarettes11 (15.9)	NA
Never smoked 6 (10.3) Ex-smoker 34 (58.6) Current smoker 18 (31.0)	Bevacizumab + paclitaxel/carboplatin	60	59.9	37 (61.7)	IIIB 5 (8.3) IV 55 (91.7)	= 0 16 (26.7) = 1 43 (71.7)	Never smoked 8 (13.3) Ex-smoker 34 (56.7) Current smoker 18 (30.0)	11 months
Smoke 493 (79)	erlotinib	617	61 (26–85)	393 (64)	IIIb 98 (16) IV 519 (84)	= 0 179 (29) = 1 358 (58) = 2 77 (13)	Smoke 472 (77)	15 months
Past or present smoker 287 (74.2) Nonsmoker 98 (25.3) Passive smoker 2 (0.5)	Sorafenib + gemcitabine + cisplatin	385	60 (28–81)	228 (59.2)	IIIB 47 (12.2) IV 338 (87.8)	= 0 146 (37.9) = 1 239 (62.1)	Past or present smoker 277 (72.1) Nonsmoker 105 (27.3) Passive smoker 2 (0.5)	NA
Never 66 (18) Former 178 (48) Current 129 (35)	Bevacizumab + Erlotinib	370	64 (31–88)	193 (52)	IIIb 32 (9) IV 317 (86) Recurrent 21 (6)	= 0 180 (49) = 1 190 (51)	Never 61 (17) Former 180 (49) Current 129 (35)	14.6 months
Never 33 (10%) Previous212 (67%) Current 72 (23%)	erlotinib plus bevacizumab	319	64.8	171 (54%)	NA	= 0 129 (41%) = 1 166 (52%) = 2 23 (7%)	Never 34 (11%) Previous 237 (74%) Current 48 (15%)	19 (0.2–34 months)
NA	bevacizumab	52	60 (38–77)	26 (50%)	NA	= 0 15 (29) = 1 30 (58) = 2 7 (14)	NA	8.1 month
Current/previous smoker 41 (79) Nonsmoker 11 (21)	Vandetanib	73	63 (27–83)	49 (67%)	IIIB 10 (14) IV 63 (86)	= 0 22 (30) = 1 51 (70)	Current/previous smoker 55 (75) Nonsmoker 17 (23)	NA

IQR, interquartile range; NA, Not Applicable. ECOG, status: Eastern Cooperative Oncology Group performance status.

The drugs analyzed in this meta-analysis include Aflibercept, Anlotinib, Axitinib, Bevacizumab, Cabozantinib, Erlotinib, Pazopanib, Ramucirumab, Sorafenib, Sunitinib, and Vandetanib. Most patients had a history of smoking, and the control groups were predominantly placebo.

### Bias risk assessment

Bias risk was evaluated using the Cochrane risk of bias tool. Most studies clearly described random sequence generation, had no incomplete data, and showed no selective reporting, thus being assessed as having a low risk of bias. Two studies exhibited incomplete outcome data and were categorized as having a high risk of bias; one also displayed selective reporting. Overall, the quality of the included RCTs was deemed high (Supplementary Figure 1).

### Network meta-analysis

**FIGURE 2:**
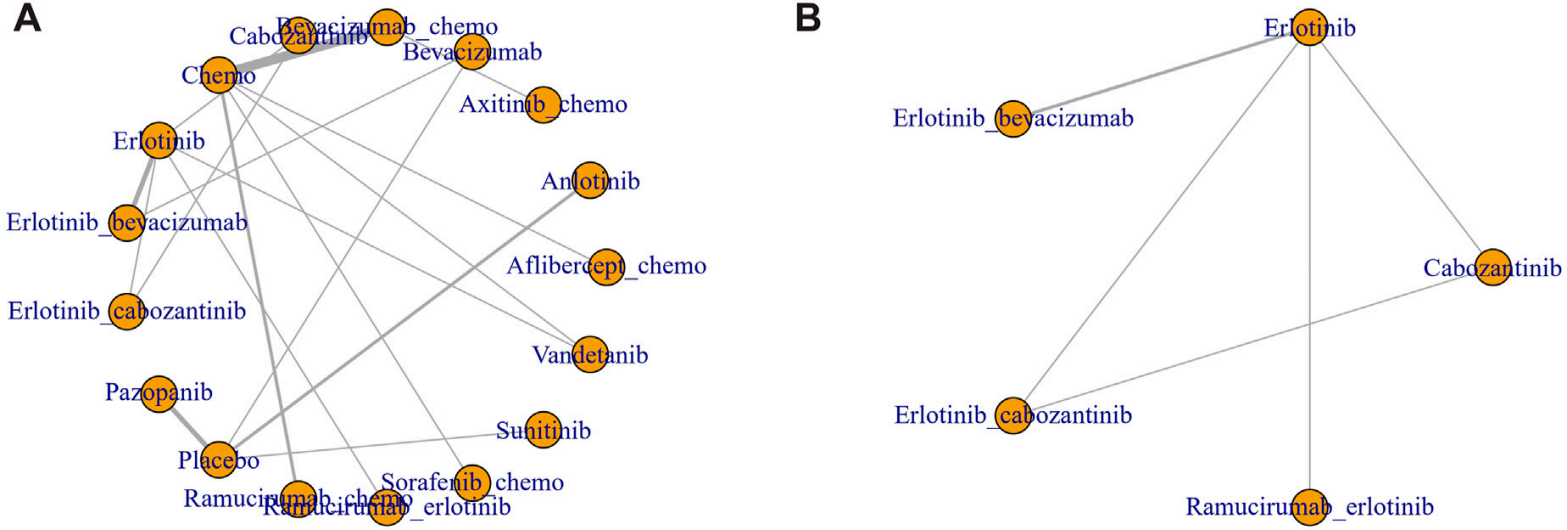
Network diagram of RCT. **(A)** Hypertension **(B)** Thrombosis. Each node represents one treatment. The size of the node is proportional to the number of participants randomized to that treatment. The edges represent direct comparisons. The width of the edge is proportional to the number of trials.

**FIGURE 3:**
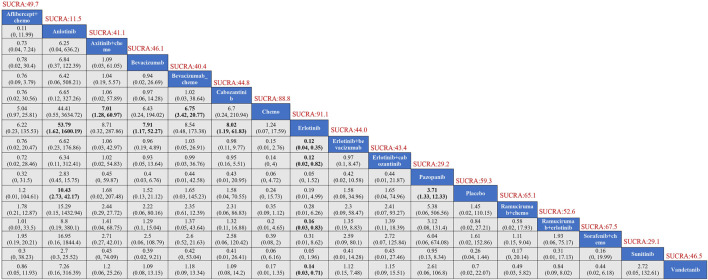
Results of TKIs compared with adverse reactions of hypertension. SUCRA, Surface Under the Cumulative Ranking Curve.

**FIGURE 4:**
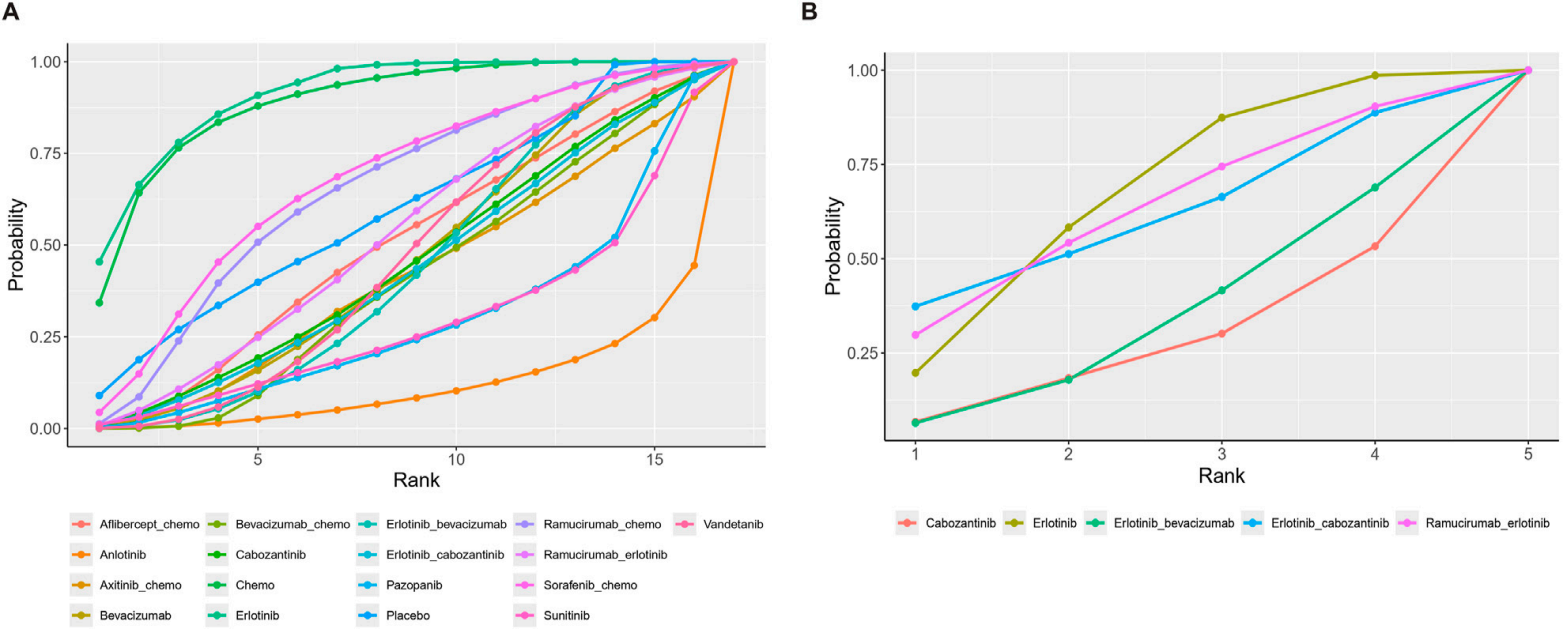
Cumulative ranking probability diagram. **(A)** Hypertension **(B)** Thrombosis. Each curve represents a treatment. The larger the area under the curve, the greater the probability of being the best treatment.

Seventeen treatment regimens were analyzed for the risk of hypertension during vascular-targeted drug therapy (Figure 2). Erlotinib exhibited the lowest risk of hypertension, with a surface under the cumulative ranking curve (SUCRA) of 91.1%. Anlotinib had the highest risk of hypertension (SUCRA = 11.5%), significantly greater than that associated with Erlotinib (HR: 53.79, 95% CI: 1.62–1600.19). Chemotherapy was the next highest in risk after Erlotinib (HR: 1.24, 95% CI: 0.07–17.59, SUCRA = 88.8%). Sorafenib combined with chemotherapy ranked third, with a risk ratio of 0.31 compared to Erlotinib (95% CI: 0.01–8.62, SUCRA = 67.5%). Axitinib combined with chemotherapy had a higher risk of hypertension compared to chemotherapy alone (HR: 1.24, 95% CI: 1.28–60.97). Cabozantinib had a significantly higher risk of hypertension compared to Erlotinib (HR: 8.02, 95% CI: 1.19–61.83) (Figure 3). The cumulative ranking probability graph in Figure 4 shows that treatments with higher SUCRA values have a lower probability of inducing hypertension, with Erlotinib, chemotherapy, and Sorafenib combined with chemotherapy being the top three treatments with the lowest hypertension risk.

**FIGURE 5:**
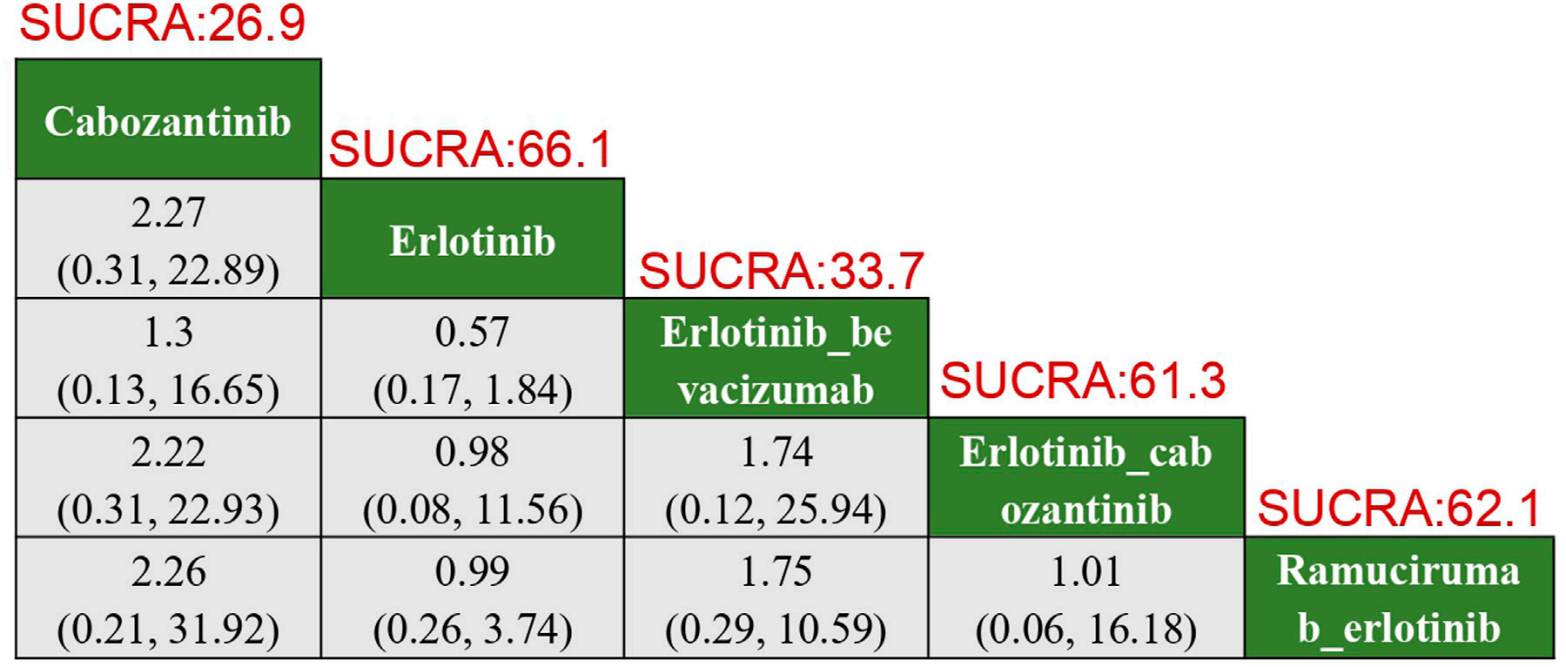
Results of TKIs compared with adverse reactions of thrombosis. SUCRA, Surface Under the Cumulative Ranking Curve.

In terms of adverse thrombotic outcomes, four RCTs were analyzed, covering five treatment regimens. Erlotinib showed the lowest risk of thrombosis, with a SUCRA of 66.0%. Ramucirumab combined with Erlotinib had the second lowest risk (HR: 0.99, 95% CI: 0.26–3.74, SUCRA = 62.1%). Erlotinib combined with Cabozantinib ranked third (SUCRA = 61.3%). Cabozantinib had the highest risk of thrombosis, with a ratio of 2.27 compared to Erlotinib (95% CI: 0.31–22.89, SUCRA = 26.9%) (Figure 5).

### Heterogeneity and sensitivity analyses

We observed moderate heterogeneity in the hypertension network (I^2^ = 45%, p = 0.03) and low heterogeneity in the thrombosis network (I^2^ = 20%, p = 0.25). Sensitivity analyses excluding high-risk-of-bias studies did not significantly alter our main findings, confirming the robustness of our results. Subgroup analyses revealed that EGFR mutation status and treatment duration did not significantly impact the relative safety rankings of the TKIs.

### Publication bias

**FIGURE 6:**
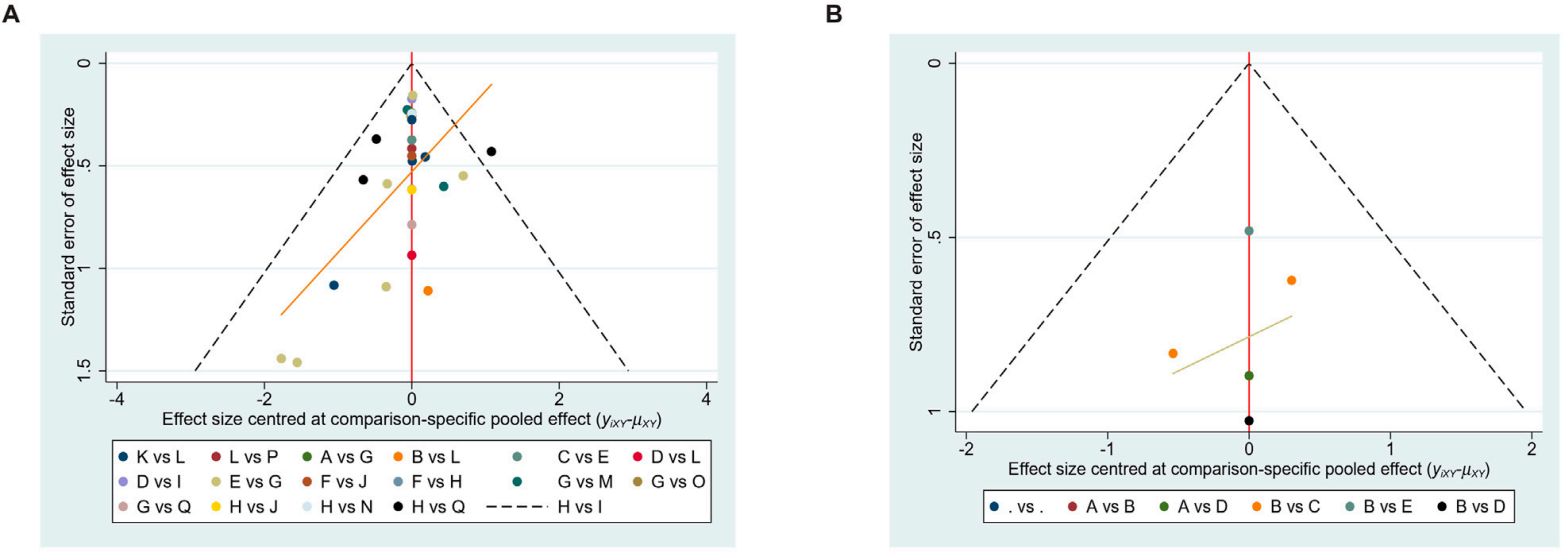
Funnel plot of network meta-analysis. **(A)** Hypertension **(B)** Thrombosis.

Funnel plots for both hypertension and thrombotic outcomes appeared roughly symmetrical (Figure 6), indicating no significant publication bias. This suggests that the results are reliable and not significantly influenced by the selective reporting of outcomes.

## Discussion

### Key findings

This study provides a comprehensive comparison of the cardiovascular safety profiles of various Tyrosine Kinase Inhibitors (TKIs) used in the treatment of Non-Small Cell Lung Cancer (NSCLC). Our network meta-analysis revealed that Erlotinib is associated with the lowest risks of both hypertension and thrombotic events among the evaluated treatments. In contrast, Anlotinib and Cabozantinib were associated with significantly higher risks of these adverse events.

To sustain their high proliferation rate, cancer cells require tumors to rapidly develop new vascular networks. However, the vasculature within tumors is often underdeveloped, which impairs its functionality (Carmeliet and Jain, 2011a). Abnormalities in tumor vascular development are partially due to irregular levels of growth factors secreted by tumor and stromal cells, with vascular endothelial growth factor (VEGF) playing a pivotal role (Carmeliet and Jain, 2011b). The poor functionality of tumor vasculature profoundly affects the tumor microenvironment, leading to hypoxia, reduced immune cell infiltration and activity, and an increased risk of metastatic dissemination. It has been proposed that antiangiogenic therapies could potentially correct these structural and functional defects in tumor vasculature (Carmeliet and Jain, 2011b; Viallard and Larrivée, 2017).

VEGF primarily interacts with two main receptors: vascular endothelial growth factor receptor-1 (VEGFR-1), also known as fms-like tyrosine kinase-1 (Flt-1), and VEGF receptor-2 (VEGFR-2). VEGFR-1 is the exclusive receptor for other VEGF family members (Papetti and Herman, 2002; Ceci et al., 2020) and is essential for hematopoiesis, matrix metalloproteinase (MMP) activation, and the migration of monocytes and other immune cells into the tumor microenvironment (TME) (Ferrara et al., 2003). In contrast, VEGFR-2 is critical for angiogenesis and vasculogenesis. VEGF binding to VEGFR-2 activates endothelial nitric oxide synthase (eNOS) and inducible nitric oxide synthase (iNOS) via the nitric oxide synthase (NOS) pathway (Zachary, 2003). This signaling pathway results in the release of vasodilators such as nitric oxide (NO), which increases vascular permeability (Lal et al., 2001). Upregulation of VEGF has been documented in various benign and malignant tumors, including melanoma, breast cancer, lung cancer, head and neck cancer, and ovarian cancer. In the tumor environment, the activation of the VEGF/VEGFR signaling axis ultimately leads to increased vascular density, invasiveness, immune evasion, and, in some cases, enhanced metastatic capacity (Jinnin et al., 2008).

The epidermal growth factor receptor (EGFR), a member of the ERBB family of cell surface receptor tyrosine kinases, is implicated in cancer progression. The binding of epidermal growth factor (EGF) to EGFR triggers phosphorylation of the receptor and other ERBB family members, leading to cell proliferation. EGFR signal transduction also contributes to tumor cell proliferation, resistance to apoptosis, angiogenesis, and metastasis (Chong and Jänne, 2013).

Recent molecular and clinical investigations have revealed intricate interactions between hypertension and VEGF signaling pathways. Specifically, hypertension-induced microvascular disruption may trigger elevated plasma VEGF expression, as evidenced by increased VEGF levels observed in patients with essential hypertension (EH) (Yang et al., 2017). This relationship appears bidirectional, with epidemiological data demonstrating significant associations between blood pressure dynamics and cancer risk (Radišauskas et al., 2016; Schairer et al., 2017).

In the context of cancer-associated complications, venous thromboembolism (VTE) emerges as a principal cause of mortality. The administration of anti-VEGF therapies has been correlated with increased VTE incidence (Posch et al., 2016), though the precise molecular mechanisms underlying this association remain to be fully elucidated. Mechanistic studies have revealed that bevacizumab administration significantly enhances plasminogen activator inhibitor (PAI-1) expression across multiple compartments, including tumor tissue, plasma, and thrombi. This observation has been further validated in mouse human lung cancer xenograft models, where bevacizumab-induced PAI-1 upregulation promotes VTE formation. Clinical validation through randomized controlled trials has consistently identified a characteristic adverse event profile associated with bevacizumab, predominantly comprising hypertension, proteinuria, hemorrhagic complications, and thrombotic events (Sandler et al., 2004).

Notably, geriatric populations demonstrate heightened susceptibility to thromboembolic and hypertensive complications during anti-angiogenic therapy (Boehm et al., 2010). This vulnerability becomes particularly relevant in the context of long-term adjuvant or maintenance treatment regimens, where the therapeutic benefits of anti-angiogenic agents must be carefully balanced against their cardiovascular risk profile.

Our analysis supports the implementation of a cardiovascular risk-stratified approach to therapeutic selection. For patients with elevated cardiovascular risk profiles, we advocate for preferential utilization of agents demonstrating superior cardiovascular safety characteristics. This strategy holds the potential to significantly reduce the incidence of thrombotic and hypertensive complications while minimizing mortality risk. Furthermore, our findings provide an evidence-based framework to guide clinical decision-making and inform the development of cardiovascular risk-adapted guidelines for targeted therapy optimization.

In this study, we evaluated these anti-angiogenic drugs to compare their risks of hypertension and thrombosis and identified the drug with the fewest side effects. Clinicians can use this information to select drugs with fewer adverse effects based on the patient’s underlying conditions, thereby improving the management of targeted therapy toxicity.

Our analysis indicates that Erlotinib has the lowest risk of both hypertension and thrombosis among the drugs studied. This conclusion was reached through constructing an indirect drug comparison network, providing highly credible evidence. Chemotherapy ranks second in terms of lowest hypertension risk. Anlotinib is associated with the highest risk of hypertension, suggesting that clinicians should carefully assess patients' baseline blood pressure and cardiovascular health before prescribing this drug. Additionally, Cabozantinib presents the highest risk of thrombosis, indicating that clinicians need to evaluate the risk of thrombosis in multiple organs and consider the prudent use of anticoagulants when administering this drug.

### Clinical implications

The clinical implications of this study are significant. In treating NSCLC, especially in patients with pre-existing cardiovascular conditions, Erlotinib should be considered as a first-line option due to its lower risk of hypertension and thrombotic events. Clinicians should exercise caution when prescribing Anlotinib and Cabozantinib, particularly in patients at high risk for cardiovascular complications. These findings underscore the importance of individualized treatment plans that weigh the benefits of tumor control against the risks of serious side effects.

Additionally, the results of this study suggest that more rigorous cardiovascular monitoring may be warranted for patients receiving high-risk TKIs, such as Anlotinib and Cabozantinib. This could involve regular blood pressure checks, thrombosis risk assessments, and the use of prophylactic measures to mitigate these risks.

### Strengths and limitations

This study has several strengths, including the use of a Bayesian network meta-analysis to integrate data from multiple studies, providing a robust comparative analysis of TKI safety profiles. The large sample size and inclusion of diverse treatment regimens enhance the generalizability of our findings.

However, several limitations of this study and their potential impacts on our findings warrant careful consideration. First, significant heterogeneity was observed across included studies, mainly due to variations in study design, patient characteristics, and outcome definitions. While our random-effects model and subgroup analyses partially addressed this issue, the heterogeneity might have led to either over- or underestimation of treatment effects, particularly in smaller subgroups.

The language restriction to English publications might have resulted in missing valuable data, particularly from Asian countries where TKIs are extensively used. This potential language bias could be especially relevant for newer TKIs that are more commonly studied in non-English speaking regions, possibly affecting our effect estimates.

The varying quality of included studies and limited long-term cardiovascular outcome data represent additional limitations. Although we conducted quality assessment and sensitivity analyses, lower-quality studies might have influenced our estimates, particularly in comparisons with fewer studies. This impact could affect our ability to fully capture the cardiovascular safety profiles of different TKIs, especially for rare adverse events.

Further prospective investigations are warranted to elucidate the cardiovascular safety profiles of combination regimens incorporating targeted therapies and immune checkpoint inhibitors, with particular emphasis on risk stratification and predictive biomarker identification.

## Conclusion

In this study, we conducted a network meta-analysis to compare the cardiovascular safety profiles of various Tyrosine Kinase Inhibitors (TKIs) used in the treatment of Non-Small Cell Lung Cancer (NSCLC). Our findings indicate that Erlotinib is associated with the lowest risk of both hypertension and thrombotic events, making it a preferred treatment option, especially for patients with pre-existing cardiovascular risk factors. Conversely, Anlotinib and Cabozantinib were found to carry significantly higher risks of these adverse events, necessitating cautious use and careful monitoring in clinical practice.

The results of this study provide valuable insights for clinicians in selecting appropriate TKIs, balancing the efficacy of cancer treatment with the potential for serious cardiovascular complications. These findings also underscore the importance of individualized treatment strategies, particularly in patients with a higher risk of hypertension or thrombotic disorders.

## Data Availability

The original contributions presented in the study are included in the article/Supplementary Material, further inquiries can be directed to the corresponding author.
