# Supplementary material for: Comparative safety profile of tyrosine kinase inhibitors in NSCLC: a network meta-analysis of hypertension and thrombotic risks
Source: Front Pharmacol. 2025 Jan 29;16:1491990. doi: 10.3389/fphar.2025.1491990 (PMC11813866; doi:10.3389/fphar.2025.1491990)

Figure S1. Quality evaluation of included literature


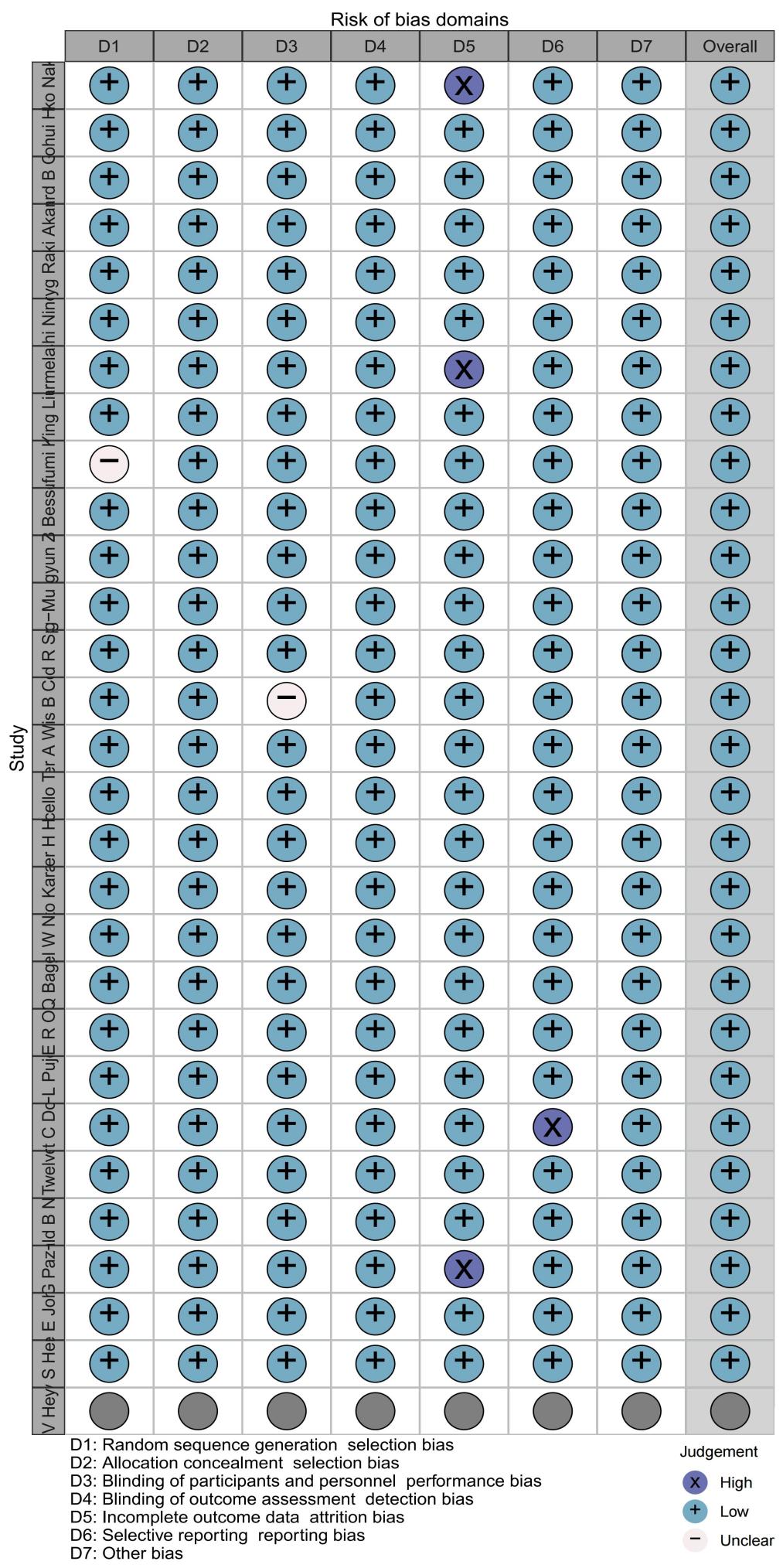


Figure S2. Comparison of hypertension outcome indicators. A. Network comparison forest plot B. Convergence diagnostic plot

A.
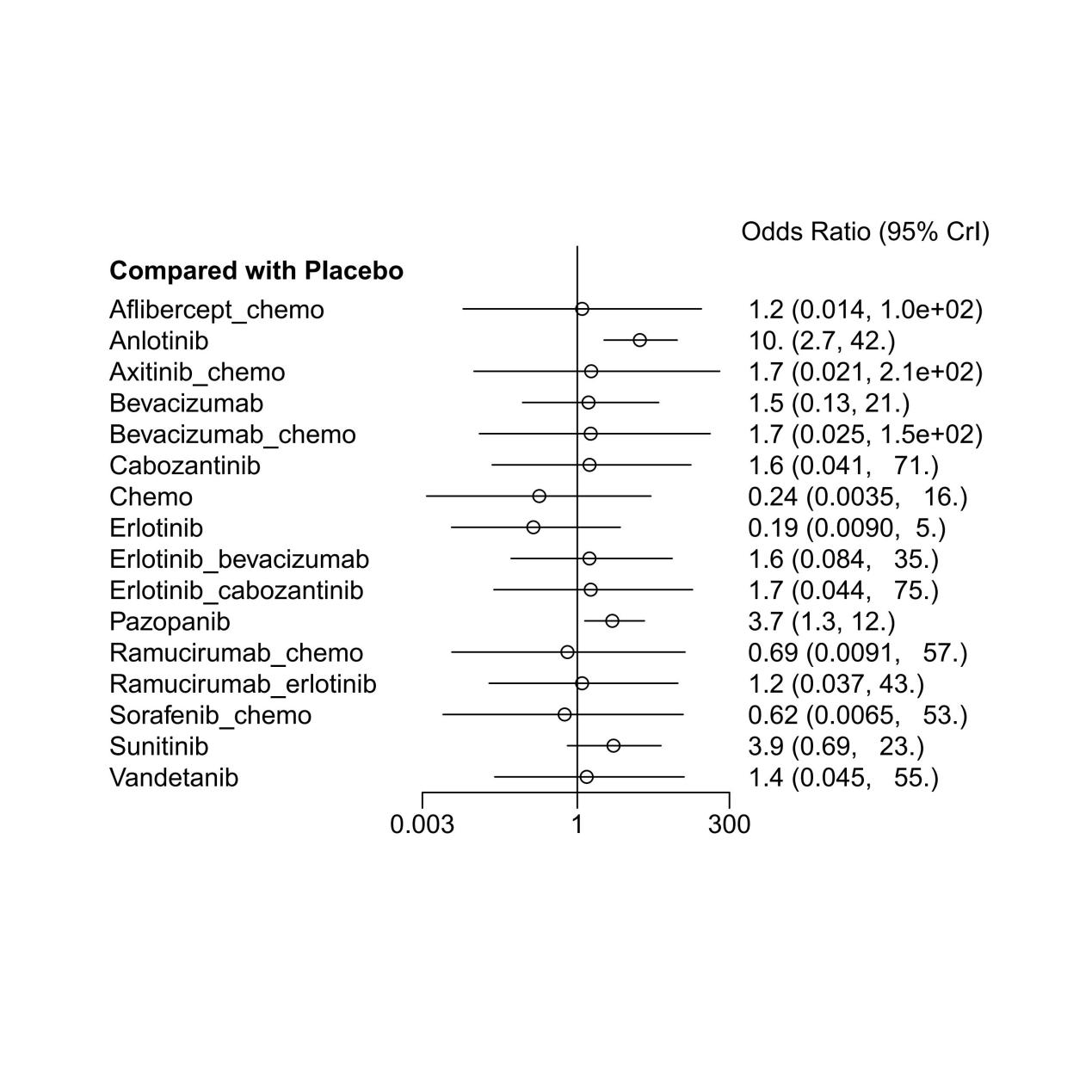


B.


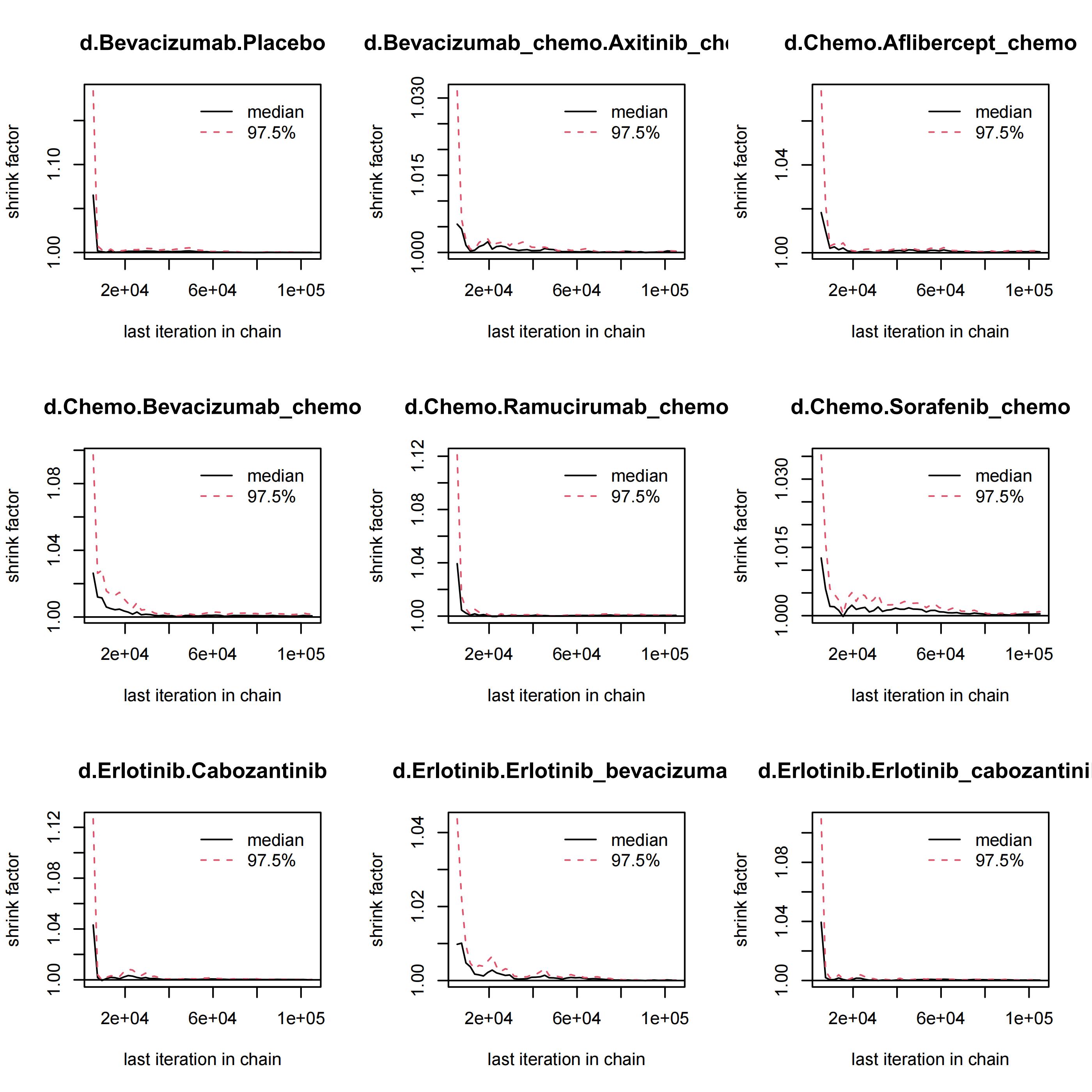


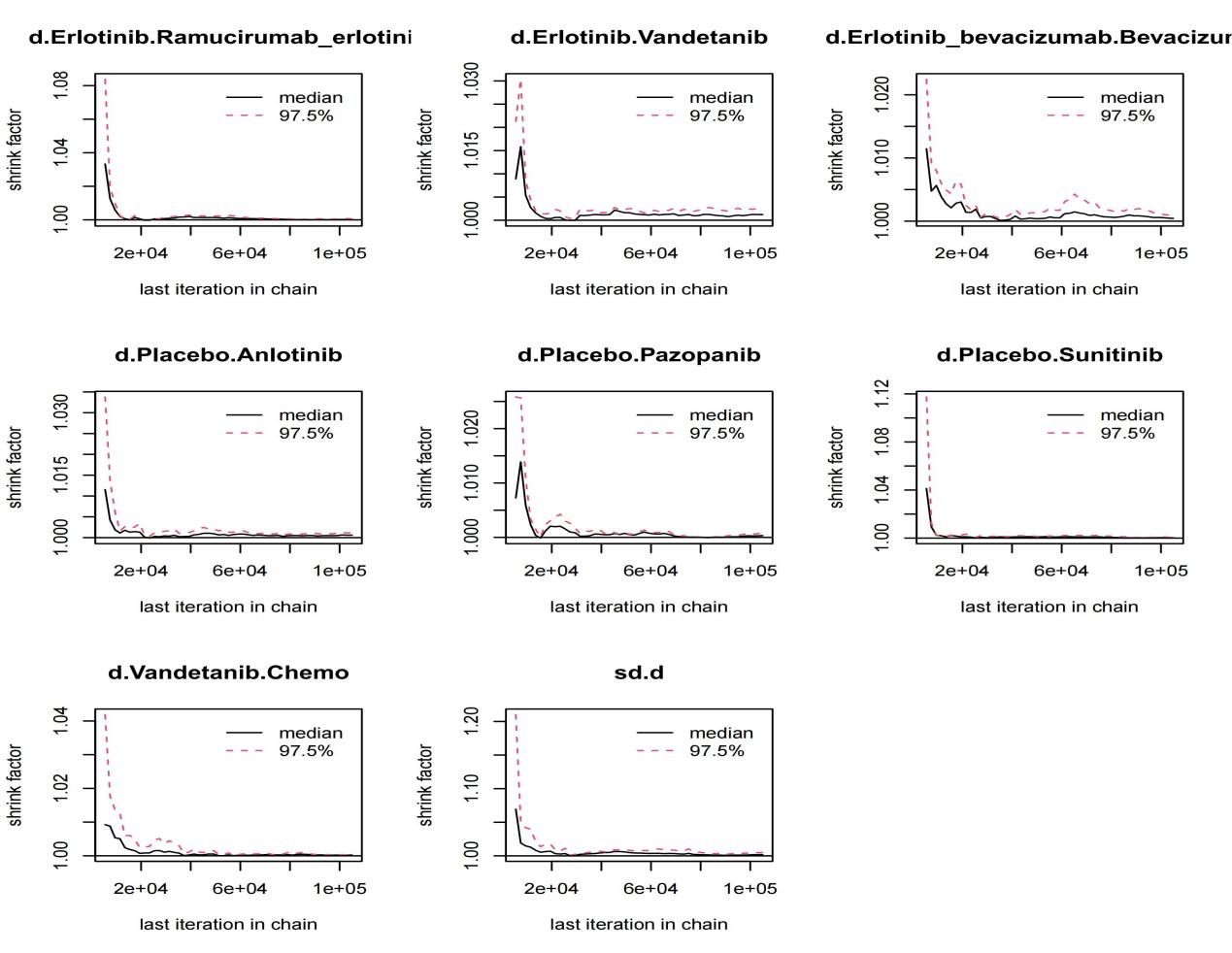


Figure S3. Comparison of embolism outcome indicators. A. Network comparison forest plot B. Convergence diagnostic plot.

A.


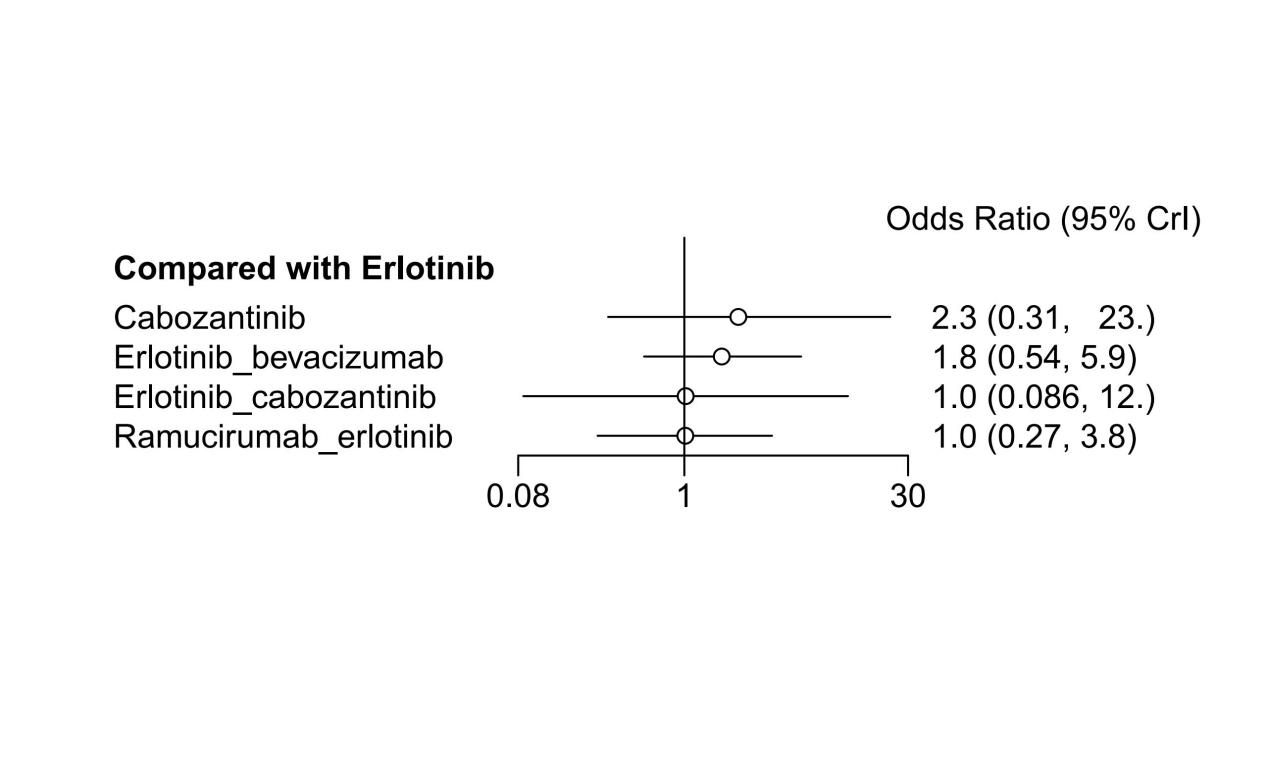


B.


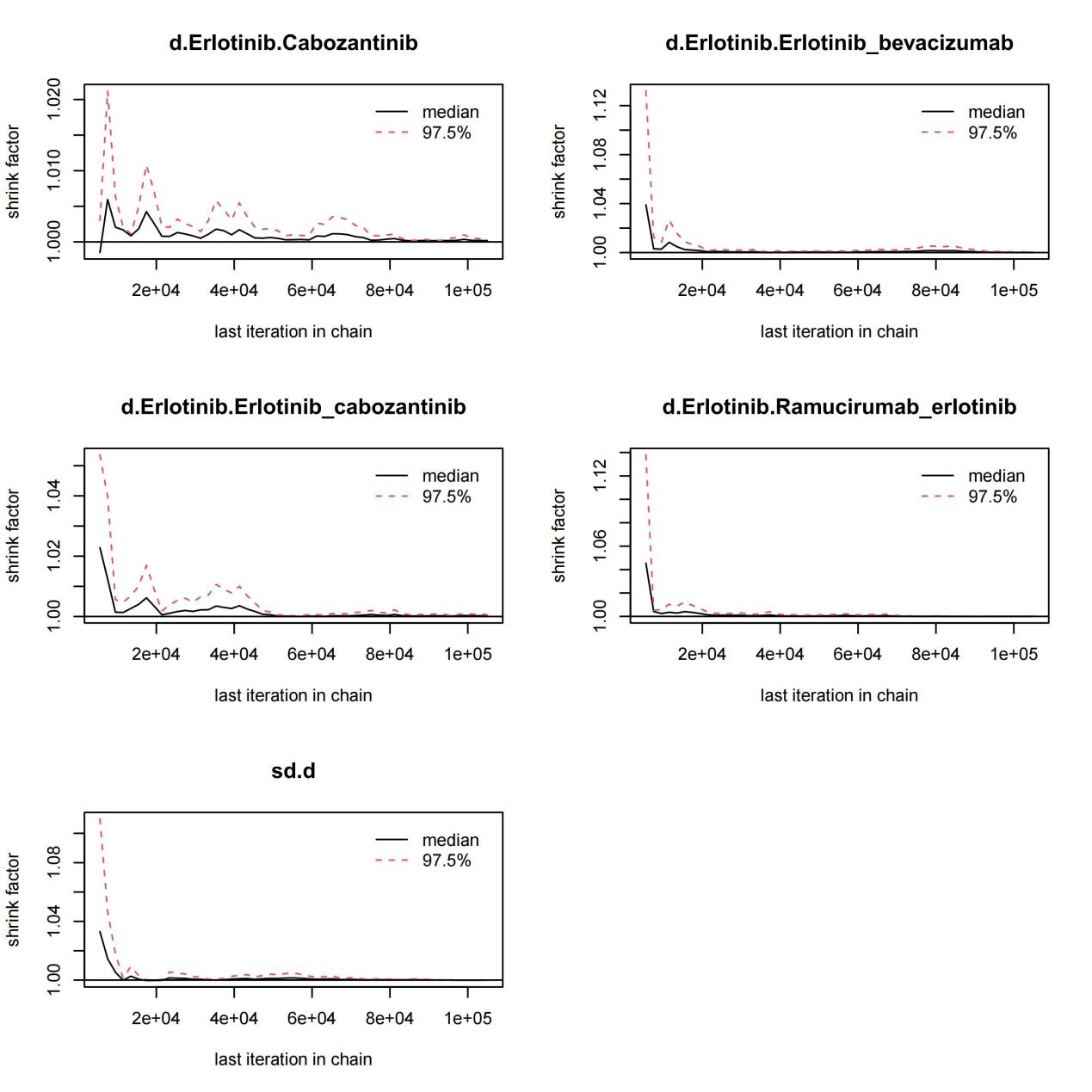

Supplement: Supplementary file 1 [file DataSheet1.docx]
